# Metastatic papillary thyroid carcinoma presenting with elevated serum levels of carbohydrate antigen 19-9 (CA19-9): a case report

**DOI:** 10.1186/s40792-022-01397-7

**Published:** 2022-03-16

**Authors:** Minoru Kihara, Akira Miyauchi, Mitsuyoshi Hirokawa, Makoto Fujishima, Hiroo Masuoka, Takuya Higashiyama, Naoyoshi Onoda, Yasuhiro Ito, Akihiro Miya

**Affiliations:** 1 Departments of Surgery, Kuma Hospital, 8-2-35 Shimoyamate-dori, Chuo-ku, Kobe, Hyogo 650-0011 Japan; 2 Department of Diagnostic Pathology, Kuma Hospital, 8-2-35 Shimoyamate-dori, Chuo-ku, Kobe, Hyogo 650-0011 Japan

**Keywords:** Papillary thyroid carcinoma, Carbohydrate antigen 19-9 (CA19-9), Liver metastasis, Thyroglobulin, Anti-thyroglobulin antibody

## Abstract

**Background:**

The major sites of distant metastases of papillary thyroid carcinoma (PTC) are the lung and bone; metastasis to the liver is rare. Although the postoperative serum thyroglobulin (Tg) level after total thyroidectomy is a good prognostic indicator for PTC when anti-thyroglobulin antibody (TgAb) is negative, the presence of TgAb interferes with the Tg assay, making serum Tg levels unreliable. Here we report a case of liver metastasis of PTC that presented with elevated serum levels of carbohydrate antigen 19-9 (CA19-9), which is usually a serum marker of pancreatic and gastrointestinal neoplasias.

**Case presentation:**

A 69-year-old man was diagnosed with PTC and underwent total thyroidectomy 16 years ago. The patient’s serum Tg levels increased progressively during follow-up and his serum TgAb was negative. Positron emission tomography (PET) and computed tomography (CT) revealed metastases of the lung, cervical spine, mediastinum and liver. The liver lesion was a solitary tumor measuring 4.0 cm in the greatest dimension. His serum CA19-9 level was very high (326 U/mL), and intrahepatic cholangiocarcinoma was suspected from the results of various examinations including gastrointestinal endoscopic imaging and CT. Laparoscopic partial liver resection for segment 4 was performed. The histopathological diagnosis was a metastatic liver tumor from PTC. The immunohistological examination revealed that the liver tumor was positive for CA19-9 and Tg. The primary PTC, recovered from paraffin-embedded specimen, was also positive for CA19-9. After the surgery, his serum CA19-9 level as well as serum Tg level markedly decreased.

**Conclusions:**

We presented the first reported case of liver metastasis of a PTC presenting with elevated serum levels of CA19-9 after total thyroidectomy. This case suggests that the serum CA19-9 levels may serve as a surrogate marker for PTC in place of the serum Tg level in patients with positive serum TgAb if the PTC and/or the metastatic lesions are positive for CA19-9 staining.

## Introduction

Papillary thyroid carcinoma (PTC) is the most prominent malignancy arising from thyroid follicular cells. However, a minority of PTC patients develop distant metastases, with the most common sites being the lungs and bones. The liver is a less common site of distant metastases of PTC [1, 2]. Thyroglobulin (Tg) is a very sensitive and specific marker in patients who have undergone total thyroidectomy for PTC. It is suggested that exponential elevation of serum Tg levels over time indicates remnant or recurrent PTC. However, the presence of anti-thyroglobulin antibody (TgAb) interferes with Tg immunometric assay and makes Tg levels unreliable. On the other hand, carbohydrate antigen 19-9 (CA19-9) is a sensitive tumor marker for pancreatic, gastric and hepatobiliary malignancies. In the present article, we report the first case of a patient with liver metastasis of PTC presenting with elevated serum levels of CA19-9.

## Case presentation

**Fig. 1:**
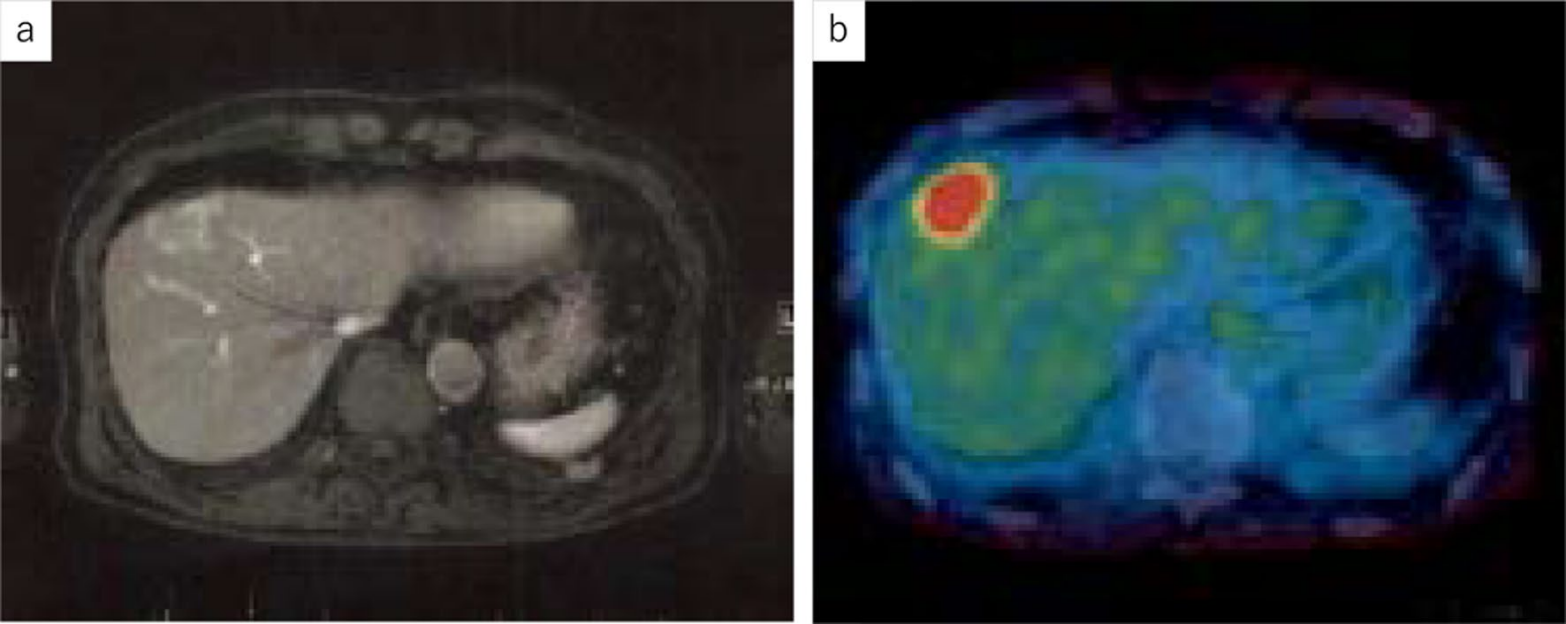
**a** Abdominal computed tomography scan showed a 40-mm solitary tumor in segment 4 of the liver. **b** Fluorodeoxyglucose positron emission tomography (FDG-PET) showed FDG uptake by the liver tumor

**Fig. 2:**
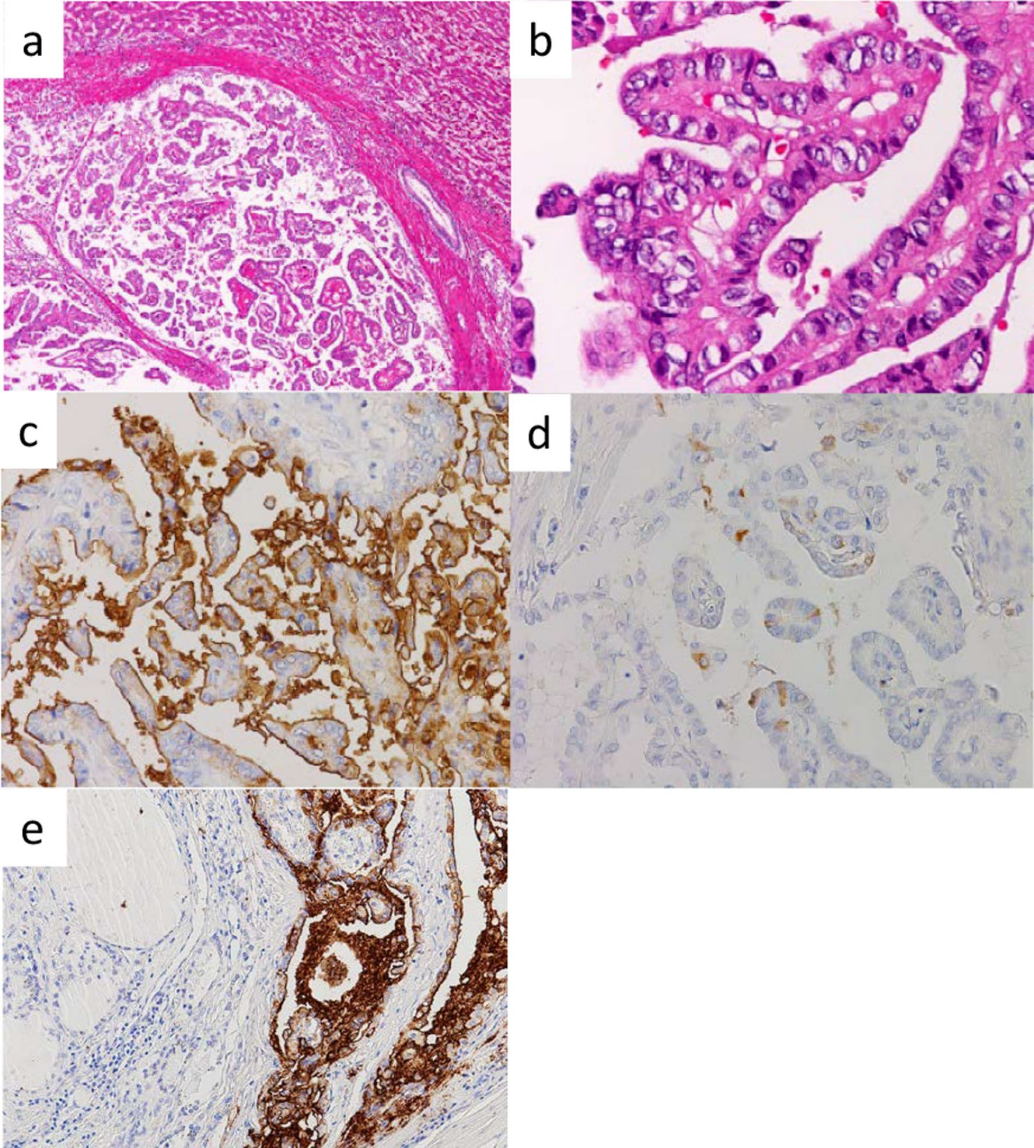
**a, b** Histopathology revealed atypical cell proliferation with a papillary structure and nuclear findings of PTC including psammoma bodies in the cells of the liver tumor. Immunohistology for the liver tumor revealed that the carcinoma cells showed intensive apical membranes positivity for (**c)** CA19-9 and along apical membranes for (**d)** thyroglobulin. **e** Primary PTC cells resected 16 years ago show cell membranous and cytoplasmic positivity for CA19-9 (right). Normal thyroid follicular cells are negative (left)

**Fig. 3:**
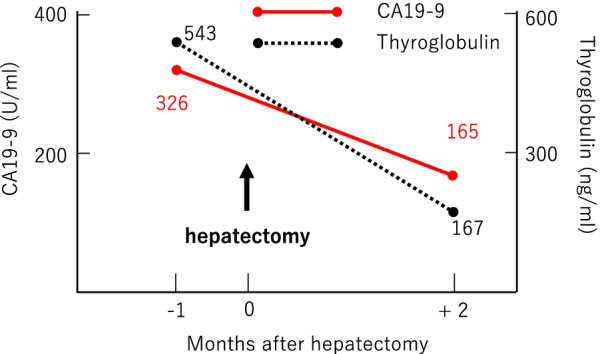
Changes in the serum levels of CA19-9 and thyroglobulin. The serum levels of CA19-9 and thyroglobulin decreased after hepatectomy

A 69-year-old man with PTC underwent total thyroidectomy, resection of the tracheal cartilage and bilateral recurrent laryngeal nerves, bilateral cervical lymph node dissection and tracheotomy at our hospital when he was 53 years. He was treated with thyroid-stimulating hormone (TSH) suppression therapy only and was followed up in the outpatient clinic of our hospital. Radioactive iodine treatment (RAI) was not performed because of a permanent tracheocutaneous fistula. About 6 years after surgery, three cervical lymph node metastases were suspected by ultrasonography. They were followed with close observation and TSH suppression therapy at the patient’s request. Serum levels of thyroglobulin antibody (TgAb) were negative. His serum Tg levels increased progressively over time during the follow-up period; therefore, positron emission tomography (PET) was performed 16 years after the surgery. It revealed ^18^F-fluorodeoxyglucose (FDG) accumulation in the liver (Fig. 1), lung, neck, and mediastinum. We considered the lung, cervical spine and mediastinal lesions as metastases of PTC, while there was a possibility of primary liver malignancy. Therefore, we referred the patient to another hospital for investigation of the solitary liver tumor measuring 4.0 cm in its greatest dimension. At this time, serum levels of CA19-9 and Tg were 326 U/mL (reference value < 37) and 543 ng/mL (reference value < 46), respectively, while his serum carcinoembryonic antigen (CEA) and α-fetoprotein (AFP) were within normal ranges. Because an intrahepatic cholangiocarcinoma was suspected from the results of various examinations including gastrointestinal endoscopic imaging and CT, a laparoscopic partial liver resection for segment 4 was performed at the other hospital. The histopathological examination revealed atypical cell proliferation with a papillary structure and nuclear findings of PTC including psammoma bodies in the tumor cells (Fig. 2a, b), with a diagnosis of liver metastasis of PTC. The patient was then referred back to our hospital for follow-up. Additional immunohistochemical examinations using the resected specimen of the liver tumor were performed at our hospital. The results showed that the tumor was positive for CA19-9 (Fig. 2c), thyroid transcription factor-1 and paired box 8 and slightly positive for Tg (Fig. 2d), while there was no staining for CEA. The final diagnosis was metastatic liver tumor originating from PTC. In addition, the primary PTC tissue resected 16 years ago, recovered from paraffin-embedded specimen, was immunohistochemically stained and found to be positive for CA19-9, while the normal thyroid tissue was negative (Fig. 2e). Two months following hepatectomy, CA19-9 and Tg levels decreased to 165 U/mL and 167 ng/mL, respectively (Fig. 3). Biopsies for metastases in the lung, neck, and mediastinum were not performed.

## Discussion

In the present case, we reported a case of a patient with liver metastasis of PTC presenting with elevated serum levels of CA19-9 after total thyroidectomy. The major sites of distant metastases from PTC are the lung and bone. However, to date, there have been few reports of liver metastases in patients with PTC [1–7]. As liver metastases are usually found along with other distant metastases sites and are nearly always multiple or diffuse [7], it is even more unusual to encounter isolated resectable liver metastases from PTC. In the present case, the patient also had metastases in the lung, cervical spine and mediastinum.

Tg is a very sensitive and specific marker in patients who have undergone total thyroidectomy for PTC; disease progression correlates with increasing serum levels of Tg in PTC patients. However, the presence of TgAb interferes with Tg immunometric assays, making the Tg levels unreliable indicators [8, 9]. The prevalence of TgAb in PTC patients is reported to be 25%, higher than that (10%) in the normal population [8, 9], and follow-up by Tg levels is not appropriate for these patients. Therefore, currently, there are no useful tumor markers to follow up on in patients who are serum TgAb-positive.

CA19-9 was originally isolated from a colorectal cancer cell line as a mucin-like product. This antigen is found in the normal epithelial cells of the gall bladder, biliary ducts, pancreas and stomach; therefore, CA19-9 is typically considered a gastrointestinal tumor marker. However, in one report, CA19-9 was observed in the tissues of ~ 6% of the patients with medullary thyroid carcinoma (MTC) [10]. Furthermore, Elisei et al. [11] and After examining 100 patients with advanced MTC, Lorusso et al. [12] reported that an elevated value of serum CA19-9 appears to be a predictive factor of poor prognosis in advanced MTC patients and could identify cases with a higher risk of mortality in the short term. Similarly, Alencar et al. [13] reported that serum CA19-9 might have a role as a prognostic factor in MTC patients. In addition, there are also a few reports on the relationship between PTC and CA19-9 [14–20]. Hashimoto et al. [14] and Vierbuchen et al. [18] found that the positive immunohistochemical staining rates for CA19-9 in PTC tissues were 58% and 48%, respectively. In one case report, serum CA19-9 showed low sensitivity as a tumor marker in a patient with thyroid carcinoma [20]; in another case report, the serum level of serum CA19-9 was elevated at the time of the growth and/or recurrence of PTC and proved a useful tumor marker [19]. Yamaguchi et al. [19] found that the serum level of CA19-9 was elevated before excision of the lung metastasis of PTC and decreased to within normal limits thereafter. In the present case, the histopathological diagnosis of the liver lesion was metastasis from PTC, and the immunostaining was positive for both CA19-9 and Tg. In addition, the primary PTC tissue resected 16 years ago was immunohistochemically stained and found to be positive for CA19-9. Furthermore, the high serum levels of both CA19-9 and Tg decreased considerably after resection of liver metastasis.

The serum levels of CA19-9 in previously reported patients with liver metastasis of PTC were normal in one patient and not described in 4 patients [3–7]. Since serum levels of CA19-9 are not usually measured in patients with PTC, the percentage of elevated serum CA19-9 levels in patients with PTC is not known. To our knowledge, high serum levels of CA19-9 in patients with liver metastasis of PTC have not been previously reported. The present report is the first reported case of a patient with liver metastasis of PTC presenting with elevated levels of serum CA19-9. If the patients with PTC have positive serum TgAb, it may be useful to immunostain the lesions for CA19-9 as a tumor marker, as in the present case. Especially, it may be a potential alternative tumor marker to Tg in PTC patients with positive serum TgAb. We recommend immunostaining the lesions for CA19-9 as a surrogate marker when the serum TgAb is positive in patients with PTC. However, of course, if the immunostaining of CA19-9 is negative in the primary thyroid lesions and metastases, the serum level of CA19-9 will not be an appropriate tumor marker.

## Conclusions

We present the first reported case of liver metastasis from PTC presenting with elevated serum levels of CA19-9 after total thyroidectomy. This case demonstrates that the serum CA19-9 levels may serve as a surrogate marker for PTC in place of the serum Tg level when both the patient’s serum TgAb is positive and immunostaining of CA19-9 of the lesions is positive.

## Data Availability

All data analyzed during this study are included in this article.

## Abbreviations

AFP: α-Fetoprotein
CA19-9: Carbohydrate antigen 19-9
CEA: Carcinoembryonic antigen
FDG: Fluorodeoxyglucose
MTC: Medullary thyroid carcinoma
PET: Positron emission tomography
PTC: Papillary thyroid carcinoma
RAI: Radioactive iodine treatment
Tg: Thyroglobulin
TgAb: Thyroglobulin antibody
TSH: Thyroid-stimulating hormone
